# Pathogenesis of COVID-19 from the Perspective of the Damage-Response Framework

**DOI:** 10.1128/mBio.01175-20

**Published:** 2020-07-02

**Authors:** Liise-anne Pirofski, Arturo Casadevall

**Affiliations:** a Division of Infectious Diseases, Albert Einstein College of Medicine, Bronx, New York, USA; b Department of Molecular Microbiology and Immunology, Johns Hopkins School of Public Health, Baltimore, Maryland, USA; University of Colorado School of Medicine; University of Texas Health Science Center at Houston

**Keywords:** COVID, viral pathogenesis, virus

## Abstract

The coronavirus disease 2019 (COVID-19) pandemic caused by the severe acute respiratory syndrome coronavirus 2 (SARS-CoV-2) presents the medical community with a significant challenge. COVID-19 is an entirely new disease with disparate clinical manifestations that are difficult to reconcile with a single pathogenic principle. Here, we explain how the flexible paradigm of the “damage-response framework” (DRF) of microbial pathogenesis can organize the varied manifestations of COVID-19 into a synthesis that accounts for differences in susceptibility of vulnerable populations as well as for differing manifestations of COVID-19 disease. By focusing on mechanisms of host damage, particularly immune-mediated damage, the DRF provides a lens to understand COVID-19 pathogenesis and to consider how potential therapies could alter the outcome of this disease.

## Abstract (precis)

The coronavirus disease 2019 (COVID-19) pandemic caused by the severe acute respiratory syndrome coronavirus 2 (SARS-CoV-2) presents the medical community with a significant challenge. COVID-19 is an entirely new disease with disparate clinical manifestations that are difficult to reconcile with a single pathogenic principle. Here, we explain how the flexible paradigm of the “damage-response framework” (DRF) of microbial pathogenesis can organize the varied manifestations of COVID-19 into a synthesis that accounts for differences in susceptibility of vulnerable populations as well as for differing manifestations of COVID-19 disease.

## INTRODUCTION

In late 2019, a new disease caused by severe acute respiratory syndrome coronavirus 2 (SARS-CoV-2) emerged in China that is now known as coronavirus disease 2019 (COVID-19). As of the time of this writing, almost seven million cases have been reported globally in a pandemic that will potentially affect billions. Despite being recognized only 6 months ago, clinical information is rapidly becoming available. COVID-19 is most severe in older individuals and those with preexisting conditions. In contrast, for most children, COVID-19 appears to be mild or asymptomatic. Although it is still early in the pandemic and much information is preliminary, there are sufficient data available to analyze the clinical manifestations and possible pathogenesis of COVID-19 in the context of the “damage-response framework” (DRF).

The DRF represents a theory of microbial pathogenesis first proposed in 1999, which views host damage as the relevant outcome of host-microbe interactions and posits that host damage stems from microbial traits or the host immune response or both (1). In the DRF, the relationship between host damage and the immune response is represented by a simple upright parabola, whereby host damage is plotted on the *y* axis as a function of the immune response, ranging from weak on the left to strong on the right (2). The DRF has been useful in advancing understanding of infectious diseases (1) and providing insight into infectious diseases that occur in the setting of weak or strong immune responses, such as candidiasis and cryptococcosis (3–5). The DRF has also informed our understanding of host susceptibility to infectious diseases (6) and can be a valuable tool for teaching microbial pathogenesis and infectious diseases (7).

According to the DRF, clinical signs and symptoms manifest themselves when host damage reaches a threshold that impairs homeostasis. In COVID-19, clinical symptoms range from a mild upper respiratory tract-like illness to a life-threatening acute respiratory syndrome (8). In the latter, oxygenation is compromised by pulmonary inflammation, a reflection of host damage. Coronaviruses damage infected cells and trigger the production of proinflammatory cytokines, which elicit inflammation that damages host cells and tissues locally and at a distance. Any inflammatory response in the lungs has the potential to cripple their primary function of gas exchange. The pulmonary failure associated with severe cases of COVID-19 is the result of lung damage caused by inflammation of the airways (9), although neurological mechanisms and thromboembolism are also contributing factors (10, 11). While virus is likely to be present in the lungs, lung damage from SARS-Cov-2 is most likely due to both viral and host factors, although their relative contributions are currently unknown.

The emergence of COVID-19 was preceded by two other outbreaks of coronaviruses in 2003 and 2012, caused by SARS-CoV-1 and Middle East respiratory syndrome coronavirus (MERS-CoV), respectively. These two viruses have been studied extensively in recent years. Until comparable information from studies of SARS-CoV-2 is available, the experience with SARS-CoV-1 and MERS-CoV provides the most important clues to the pathogenesis of the severe coronavirus-related disease caused by COVID-19. Like COVID-19, severe disease in both SARS and MERS involved respiratory failure. Analysis of viral burden in those with the most severe disease showed that their viral titers were declining (12). This is consistent with the concept that an overexuberant immune response contributed to lung damage. Experiments in animal models showed that highly human-pathogenic coronaviruses such as the agents of SARS and MERS exhibit rapid viral replication in the lungs that triggers a proinflammatory cytokine response that in turn elicits an inflammatory cell infiltrate that damages the lung (13). Hence, for SARS and MERS, lung damage that affected pulmonary function and host survival had a strong component of immune-mediated damage. Although only a few immunocompromised patients have been reported in the SARS and MERS literature, two cases suggest that the disease may have been milder in those with impaired immunity, perhaps supporting the idea that lung damage follows a strong immune response. A patient with AIDS was reported to have a mild case of SARS, a fact attributed to the weakened condition of the immune system of the patient having resulted in a lack of severe disease (14). An early case report describing SARS-CoV-2 infection in persons with HIV infection did not compare the severity of the disease to that seen in HIV-uninfected persons (15). Notably, a bone marrow transplant recipient on immunosuppressive therapy suffered only mild SARS after infection with SARS-CoV-1 (16). These reports support the idea that exuberant inflammation may be the primary driver of lung damage in patients with COVID-19. While this concept requires further investigation as there are currently no data on the course of COVID-19 in immunocompromised patients, it is consistent with findings in fatal cases during the H1N1 pandemic in which inflammatory damage in the lungs occurred in the absence of significant viral particles (17). On the other hand, older age and low lymphocyte counts, as well as elevated levels of inflammatory markers, were associated with the development of acute respiratory distress syndrome in patients with COVID-19 in Wuhan, China (18). Although the relationships among immune suppression, the inflammatory response, and lung damage in COVID-19 remain to be unraveled, the link between elevated inflammatory markers and respiratory failure has led to the use of immunosuppressive agents ranging from corticosteroids to inhibitors of cytokines, such as interleukin-6 (IL-6) and other inflammatory mediators (19).

## COVID-19 CLINICAL AND EPIDEMIOLOGIC FEATURES

The rapidly evolving clinical picture of COVID-19 presents a series of observations that include the following:

1. Prevalence and severity of the disease increase with age.
2. Many if not most infections are asymptomatic or produce only mild disease.
3. Asymptomatic infected individuals can transmit SARS-CoV-2, implying viral shedding.
4. Individuals with cardiovascular disease, diabetes, obesity, diabetes, and pulmonary disease have higher mortality.
5. COVID-19 disease is less severe and may be asymptomatic in children, but a small minority develop a multisystem inflammatory syndrome with similarities to Kawasaki’s disease.
6. Sudden deterioration with progressive pulmonary failure can occur in younger individuals.
7. The incidence and severity of disease are higher in men than in women.
8. Death is usually the result of anoxia due to pulmonary failure, although there is increasing evidence of dysfunction in other organs, including heart, kidneys, and tissues affected by dysregulated coagulation and thrombosis.

## COVID-19 FROM THE PERSPECTIVE OF THE DRF

The DRF framework posits that host damage can occur in the setting of either a weak or a strong immune response. An important caveat when considering the strength of the immune response and disease in the context of the DRF is that strong immune responses are not necessarily protective if they damage tissue by inducing exuberant inflammatory responses, irrespective of whether they clear the microbe. Thus, considering host damage as a function of immune response is a conceptual approach that provides a roadmap for incorporation of viral load and host inflammation into an understanding of the clinical manifestations and pathogenesis of COVID-19.

The observation that many SARS-CoV-2 infections are asymptomatic or result in mild disease implies that the immune response in such individuals controls the virus without eliciting damage that translates into clinical manifestations. In contrast, the manifestations of COVID-19 that occur in aged persons and/or those with pulmonary, cardiac, and endocrine conditions are likely to reflect a different immune response that may result in a degree of damage that increases disease severity. The immune response of those with comorbidity conditions may be weaker and less effective in clearing virus. Aging is known to be associated with weaker immunity (6). Similarly, pulmonary disease, cardiac disease, and endocrine disease are each associated with organ and/or tissue damage that can impair local immune responses and increase the likelihood of viral dissemination and progressive disease. Men are also known to be more vulnerable to many infectious diseases than women due to stronger immune responses in women (20). Hence, weaker immune responses might explain the observation that COVID-19 is more severe in the aged, the chronically ill, and men.

The relative rarity of symptomatic COVID-19 disease in children infected with SARS-CoV-2 (21) is striking. A report from China revealed that 79% of children either were asymptomatic or had mild disease (22), and in Italy, only 12% of children seen in emergency departments with positive tests for SARS-CoV-2 were ill (23). The mean age of these children was 3.3 years. This is notable since young children are often more susceptible than adults to respiratory viruses that cause pneumonia such as influenza and respiratory syncytial virus. The increased susceptibility of infants and young children to infectious diseases is often attributed to their having immature immune systems, as exemplified by their inability to mount antibody responses to polysaccharides before age 2. However, MERS was often asymptomatic in children, except those who were immunocompromised (24). Similarly, children were found to be susceptible to but less likely to have severe disease with SARS-CoV-1 (25). Thus, the immune response of children to these betacoronaviruses may differ from that of adults. It has been hypothesized that one factor contributing to the relative paucity of severe disease in children may be a higher percentage of CD4 T cells (26). This is consistent with the idea that the immune systems of children may contain SARS-CoV-2 more efficiently, leading to less damage. However, a study in Wuhan found that infants and younger children were more likely to have severe disease than older children (22), suggesting that age-related differences in the immune response may play an important role in containment of SARS-CoV-2. It is also possible that children have a different distribution of angiotensin converting enzyme 2 (ACE2), the receptor for SARS-CoV-2 (27). Another possibility is that children’s immune systems do not react with a degree of intensity that causes tissue damage, because they have not experienced multiple infections with other endemic human coronaviruses. If this is the case, the more severe disease observed in older individuals could reflect exuberant immune responses fueled by immunological memory of prior coronavirus infections. However, some children infected with SARS-CoV-2 develop an inflammatory vasculitis known as multisystem inflammatory syndrome in children (MIS-C) with features resembling those associated with Kawasaki’s disease (28). Although this syndrome has been described only recently and little is known about its pathogenesis, the clinical findings are those of a severe vasculitis triggered by inflammation, which again implicates strong immune responses in another facet of COVID-19 disease.

The finding that most SARS-CoV-2 infections are asymptomatic and that asymptomatic individuals can transmit the virus to others has important implications for the pathogenesis of this disease. The issue of asymptomatic transmission of other respiratory microbes remains a subject of intense study (29, 30). The presence of high levels of SARS-CoV-2 in the upper respiratory tract is believed to explain the efficiency of asymptomatic transmission (31). Transmission by persons without symptoms means that SARS-CoV-2 replicates to a level sufficient to be acquired by another person without damaging host tissues sufficiently to trigger clinical signs and symptoms. This also implies that for some individuals, repeated cycles of viral replication do not translate into damage that results in symptoms. Notably, SARS-CoV-2 antibody levels were lower in hospitalized patients with less-severe disease, suggesting that there is more to learn about relationships among viral load, the immune response, and disease manifestations (32).

The main clinical manifestation of severe COVID-19 is pulmonary deterioration. This often occurs in individuals with high levels of proinflammatory cytokines, in whom a “cytokine storm” is believed to drive disease pathogenesis. In support of this hypothesis, patients with more-severe COVID-19 had higher serum levels of IL-2R, IL-6, IL-10, and tumor necrosis factor alpha (TNF-α) than patients with milder disease (33). However, they also had lymphopenia, suggesting the presence of cytokine storm may reflect a failure of cellular immunity to contain the virus. The association of cytokine storm with severe disease and mortality has led to the use of various immunosuppressive agents in patients with elevated inflammatory markers (34). Although it is too early to assume that the association of elevated inflammatory markers with severe COVID-19 implies causation, clinical deterioration in the setting of a strong proinflammatory response is consistent with host damage from an overexuberant immune response.

In summary, COVID-19 is more severe in people who are likely to have impaired immunity, such as the aged and/or those with comorbidity conditions (35). This supports the concept that in such individuals, weak or impaired immune responses may predispose to the development of disease. Consistent with this hypothesis are data associating lymphopenia and low T cell levels (36) with severe disease and the observation that the prevalence of disease is lower in women, who mount stronger immune responses than men. In contrast, the association of high levels of proinflammatory mediators and cytokine storm with disease severity (37); histologic findings of inflammation, an inflammatory prothrombotic state; and an absence of viral inclusions in some postmortem reports (38–40) suggest that the immune response is itself a major contributor to host damage in COVID-19. In this paradigm, the asymptomatic to less-severe responses of younger people without comorbidities and of children may reflect a “balanced” immune response that clears virus without inducing inflammatory damage.

## A SYNTHESIS FOR COVID-19 PATHOGENESIS

**FIG 1:**
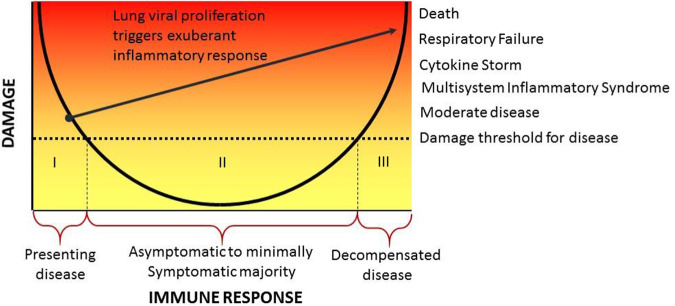
Pathogenesis of COVID-19 in the context of the DRF. The clinical manifestations of COVID-19 suggest there are three types of individual responses to SARS-CoV-2 infection (indicated as “I,” “II,” and “III” in the figure). Group I consists of individuals who mount an inadequate response to infection and cannot control the virus. For some such individuals, this leads to viral proliferation that triggers an exuberant inflammatory response (line leading from group I to group III). Group II consists of individuals who are asymptomatic or minimally symptomatic after infection with immune responses that effectively control the virus without mediating sufficient host damage to impair homeostasis. Group III consists of individuals that either mount an initial tissue-damaging response to SARS-CoV-2 infection or suffer progressive inflammatory damage as a result of unchecked viral replication in their lungs.

The DRF provides the ideal paradigm to delve deeper into the pathogenesis of COVID-19 by placing its different clinical manifestations on the axes of a parabola to account for their occurrence as a function of host susceptibility and the immune response (Fig. 1). In this schema, individuals are categorized into three groups. Group I consists of individuals with comorbid conditions that may impair the immune response. The response of these individuals is located on the left side of the parabola, indicating they may develop higher viral burdens and may be at greater risk for progression to severe disease due to an inability to contain the virus in the nasopharynx. In some such individuals, unchecked viral proliferation may trigger an excessive inflammatory response that further damages the lungs and progresses to pulmonary failure. This would indicate a transition to group III (see below). In some, progressive disease is also characterized by hematological dyscrasia, coagulation abnormalities, and multiorgan failure, which may stem from immune dysregulation. In contrast, those who do not progress to pulmonary failure may be able to mount an effective response that eventually contains the virus, leading to recovery. Group I also includes immunologically intact individuals who acquired a large inoculum that overwhelmed local defenses, resulting in a *de facto* weak immune response. Group II consists of individuals who are infected with SARS-CoV-2 and remain asymptomatic or manifest only mild disease. For such individuals, the immune response contains the viral inoculum and subsequent viral replication occurs without leading to a degree of damage that alters homeostasis. These individuals have mild or no symptoms of COVID-19, although they may transmit virus to others. This group also includes children, most of whom exhibit mild to no disease manifestations, and possibly some pregnant women who may also be asymptomatic (41). Group III consists of individuals from group I whose decompensated disease outcome is the result of an excessive immune response as well as individuals who mount an excessive initial inflammatory response to infection, resulting in cytokine storm, pulmonary failure, progressive organ damage, and death.

The mode of infection for SARS-CoV-2 is generally accepted to be introduction through the respiratory tract. Diagnosis is made by PCR detection of viral nucleic acids in the upper respiratory airways, usually the nasopharynx. At this time, definitive statements about the relationships between nasopharyngeal virus, pneumonia, and disease are difficult to make because the PCR test may be associated with false-negative results (42). With this caveat, we note that asymptomatic group I individuals may be positive for nasopharyngeal virus (43, 44). Those in groups II and III manifest clinical signs and symptoms of disease that range from cough to respiratory failure. At this time, the temporal relationship between nasopharyngeal infection and pneumonia is not well understood, but it is clear that in some individuals upper airway infection precedes pneumonia, while in others there is radiographic evidence of pneumonia with negative nasopharyngeal PCR test results (45). The relationship between nasopharyngeal virus and pneumonia may vary depending on the site of initial infection. Infection from large droplets that settle in upper airways or fomites could lead to initial nasopharyngeal infection that subsequently spreads to the lungs. On the other hand, infection from small droplets that reach alveoli would produce lung-first infection that could subsequently reach the upper airways. The relationship between the initial infection site in lung or nasopharyngeal sites and outcomes in groups I to III is unknown, as progression to disease would depend on rates of viral replication and the nature of the immune response.

**FIG 2:**
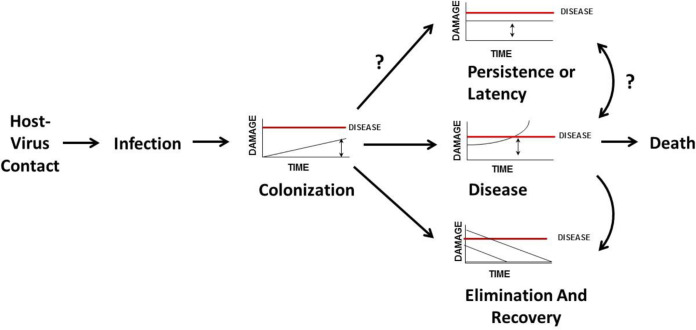
The states of COVID-19 infection. Considering damage as a function of time, the DRF posits that SARS-CoV-2-human host interaction results in the states of colonization or disease, which can lead to viral elimination and recovery or possibly to latency. A state of persistence or latency has not been associated with SARS-CoV-2 infection, although, theoretically, it could exist.

Another way to apply the DRF to COVID-19 is to consider host damage as a function of time. Using this approach, we can organize the outcome of host-virus interaction into discrete states associated with known outcomes of SARS-CoV-2 infection (Fig. 2). The DRF defines four states to describe the outcome of host-microbe interaction: commensalism, colonization, persistence (latency, chronicity), and disease (2, 46). The states of colonization and commensalism are the same when the amount of damage from the host-microbe interaction is nil. Latency is a recognized state that follows infection with some viruses. Although “colonization” is not a term commonly used to describe viral infections, use of the term in this context helps us to define the outcome of host-virus interaction and to characterize host damage as a function of time. For SARS-CoV-2, we can think of the time that the virus is replicating in the nasopharynx of an infected individual as representing a state of colonization. During that period, the amount of damage that the host experiences is below the threshold that translates into symptoms. Thus, the person is asymptomatic and yet is able to transmit virus, i.e., is in a contagious state. This is the reason that the CDC recommended that all persons wear masks in public (47), namely, to lessen the chance that an asymptomatic person would transmit the virus to others. Colonization can elicit an immune response that leads to SARS-CoV-2 elimination and resolution of infection or a response that leads to progressive host damage and disease. Disease leads to death when host damage is irreparable and compromises vital organ function. At this time, we do not know if there is a chronic persistent state that would correspond to the state of latency.

## PARALLELS TO OTHER PULMONARY INFECTIOUS DISEASES

An important tenet of the DRF is that strong immune responses do not necessarily represent effective immune responses. In fact, they can be deleterious to the host by mediating inflammatory damage if they eradicate the microbe or if they fail to do so. To make this point for COVID-19, we consider parallels between *Pneumocystis* pneumonia (PCP) in patients with HIV-AIDS and in patients with comorbidities and COVID-19. For both COVID-19 and PCP, severe disease is accompanied by exuberant pulmonary inflammation that impairs gas exchange and is life-threatening. In PCP, severely immunocompromised individuals develop a progressive pneumonia associated with hypoxia, pulmonary failure, and death as alveolar inflammation compromises oxygenation. Addition of corticosteroid therapy significantly improves survival of HIV-infected persons with hypoxia due to PCP (48). Initially, the use of corticosteroids in PCP was frowned upon because it involved giving an immunosuppressive drug to patients with profound immunosuppression. Today, the beneficial use of corticosteroids in PCP is a standard of care per the following schema: HIV-associated immune impairment → replication of *Pneumocystis* spp. in the lungs → increase in levels of fungal antigens → florid immune response → pneumonia → impaired oxygenation. We propose that a similar sequence of events may occur in severe COVID-19 in those with preexisting cardiovascular and pulmonary disease and the aged, except that there is an increased viral burden instead of an increased fungal burden. Like the intense inflammation seen in cases of HIV-associated PCP, the extensive lung inflammation in patients with COVID-19 who have conditions associated with impaired immunity may seem paradoxical. However, it is worth noting that impaired immunity includes dysregulated immune responses that are often characterized by chronic and/or inappropriate inflammation. Hence, as noted above, individuals in group I include those who mount a weak and/or inadequate response to SARS-CoV-2 infection that leads to immune responses that result in transition to the type of damage seen in individuals in group III (as indicated by the line in Fig. 1).

Another disease with a clinical course that has some parallels to COVID-19 is pneumococcal pneumonia. In the preantibiotic era, descriptions of this disease noted progressive symptoms with a “crisis” at 7 to 8 days that was associated with the appearance of serum antibody and agglutinins, that was temporally associated with defervescence and recovery (49, 50). In contrast, an inability to mount an effective antibody response was associated with extrapulmonary dissemination and a systemic inflammatory syndrome that often led to death. The clinical course of COVID-19 may also have a turning point after an initial phase of fever and cough that is followed by either clinical improvement or deterioration that can progress to pulmonary failure. The mediator(s) and/or correlate(s) of this turning point in COVID-19 are not currently known. The “turning point” of COVID-19 has parallels to SARS1, as well as to 2009 pandemic H1N1. In those diseases, the immune system either eliminated the virus without triggering damage, resulting in an asymptomatic state, or triggered inflammation and damage, resulting in respiratory failure.

Severe COVID-19 and other pulmonary infectious diseases, such as *Pneumocystis* infection, pneumococcal disease, and influenza (H1N1) virus and SARS1 virus pneumonia, are each notable for clinical manifestations that are largely driven by inflammatory damage despite being caused by a fungus, bacterium, and viruses, respectively, and, for pneumocystis infection, pneumococcus disease, and influenza, the availability of effective antimicrobial therapy. These parallels highlight the ability of the DRF to deconstruct microbial pathogenesis in a way that illuminates the role of the host in mediating damage that translates into clinical manifestations. Hence, for COVID-19, like the other pulmonary diseases, the inflammatory response makes a critical contribution to host damage, and avoidance or abrogation of this damage should be an important component of therapeutic strategies.

## THERAPEUTIC APPROACHES TO COVID-19

Considering the pathogenesis of COVID-19 through the lens of the DRF provides a roadmap for therapeutic approaches to this disease. The therapeutic goal is to reduce host damage, which would reduce the prevalence and severity of disease and translate into reduced mortality. Currently, there are no validated therapies for COVID-19, but many are under development and investigation. Therapeutic approaches can be broadly placed into three groups depending on whether the primary goal is to target the virus or the host immune response or both, with the important caveat that antiviral and anti-inflammatory effects are interdependent such that a strategy that targets one is likely to also affect the other.

**(i) Antiviral.** The ability of small molecules that interfere with HIV, herpesvirus, and hepatitis virus replication to treat diseases caused by these viruses established the principle that drugs that target the virus can be effective in viral diseases. Today, numerous compounds are under investigation that target viral replication and there is confidence that effective antiviral compounds against SARS-CoV-2 will be developed. From the viewpoint of the DRF, these drugs function by reducing viral burden, which can reduce virus-mediated tissue damage and avert or reduce the development of organ-damaging inflammatory responses. One such drug is remdesivir, an antiviral agent that received emergency authorization for use in COVID-19 (51). Although the effect of remdesivir on SARS-CoV-2 viral load in patients has not been published, it ameliorated disease in MERS coronavirus-infected macaques by inhibiting viral replication (52) and was recently shown to reduce lung viral loads in macaques (66).

**FIG 3:**
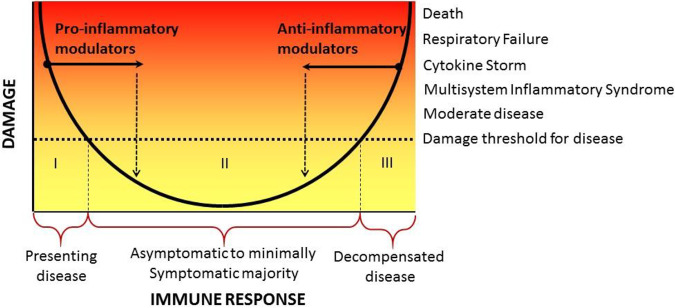
Effect of immune modulators on the position of an individual on the DRF parabola. For those with weak immune responses (left side of the *x* axis), treatment with an immune modulator that enhances immunity may move them to a position to the right representing reduced damage. Conversely, for those with disease resulting from immune-mediated host damage (right side of the *x* axis), treatment with an immune modulator that diminishes the intensity of the immune response may move them to a position to the left representing reduced damage.

**(ii) Immunomodulator.** This class of agents modifies the course of disease by affecting the immune response and moving the individual’s immune response to either the right or the left along the *x* axis of the DRF parabola (Fig. 3). Given what we know about the pathogenesis of COVID-19, immune modulators that enhance the immune response would be contraindicated, although one can imagine potential usefulness in individuals at the extreme left of the parabola, if they are unable to mount an effective antiviral response. In this regard, we note that although interferons are critical for an effective immune response against coronaviruses, their therapeutic role in experimental and clinical disease is uncertain and could be detrimental (53), possibly because we currently cannot identify the group of patients on the left of the parabola who would benefit from this intervention. On the other hand, there are ongoing clinical trials with anti-inflammatory drugs targeted to various inflammatory mediators, such as IL-6, including tocilizumab and high-dose intravenous gamma globulin (54). The idea behind the use of these agents is to inhibit the development of damage mediated by the host inflammatory response. Targeting inflammation proved successful for *Pneumocystis* pneumonia. Some studies support the use of corticosteroids in bacterial pneumonia, and there is anecdotal evidence for the efficacy of immune modulators such as statins in viral pneumonias (55, 56). Steroids are used widely for COVID-19 and a press release from a large randomized clinical trial (RECOVERY) reported that they reduced mortality in patients requiring oxygen supplementation (https://www.recoverytrial.net/files/recovery_dexamethasone_statement_160620_v2final.pdf). From the viewpoint of the DRF, immune modulating agents such as corticosteroids reduce inflammatory damage and shift the curve to the left (Fig. 3). Although immune modulating agents do not target the virus directly, the hope is that by reducing host damage the host will gain time to mount effective antiviral responses that lead to viral elimination and recovery. However, given the cascade of inflammation that may extend to complement and noncytokine/nonchemokine mediators, the use of two or more types of immune modulator might be necessary to harness and extinguish SARS-CoV-2-mediated immune dysregulation. The concern about the use of steroids and other agents that suppress the immune response in COVID-19 is that they may inhibit antiviral activity and/or increase the likelihood of superinfection with bacteria and fungi. Clinicians must be vigilant and aware of the latter. The ability to identify those patients who would most benefit from moving to the left on the DRF parabola would help clinicians administer such agents with more confidence.

**(iii) Specific immunoglobulins.** The only compounds that reliably target both the virus and the immune response are specific immunoglobulins, as they have both virus-specific neutralizing and protean effects on the immune response ranging from activation of Fc receptors and complement to enabling antibody-dependent cellular cytotoxicity. There is an extensive body of evidence supporting the efficacy of convalescent plasma to treat viral pneumonias that dates back to the 1918 epidemic. Although most reports, case series, and uncontrolled studies have been favorable (57–59), the approach has not been tested in large randomized controlled trials. We have proposed that this approach be considered and tested (60, 61), and there is some evidence for its usefulness against COVID-19 (62–65).

## APPROACH TO THE PATIENT

Analysis of COVID-19 patients in the context of the DRF suggests that the optimal therapeutic approach should depend on where their immune response lies on the parabola. Although all patients with COVID-19 may benefit from antiviral agents that target SARS-CoV-2, optimal therapy is likely to also require agents that reduce host damage by modulating the immune response and moving the patient’s position to the left along the *x* axis. Treatment with immune modulating drugs may be important for those individuals with either insufficient or overexuberant immune responses that result in severe damage and disease. The DRF suggests a way to identify patients for clinical trials of investigational therapies, particularly for agents that affect the immune response. Clearly, individuals with COVID-19 in groups I and III are likely to respond differently to immune modulating drugs even when presenting with similar clinical conditions (7). As we gain a greater understanding of the way in which the clinical manifestations of COVID-19 reflect viral factors and the host immune response, it will be possible to develop algorithms that place affected individuals on the parabola to guide and optimize approaches to therapy.

## SUMMARY: COVID-19 IN THE CONTEXT OF THE DRF

The DRF makes it possible to assemble the diverse manifestations of COVID-19 into one synthesis that explains the pathogenesis and clinical outcomes of this disease as a function of the host immune response. The DRF also provides a schema to design therapy based on anticipated effects on disease pathogenesis and host response and how they affect the position of the patient on the *x* axis of the DRF parabola. The DRF is flexible and will be able to accommodate new information as we learn more about COVID-19. For example, between the date when this paper was first submitted for publication and its revision, MIS-C, which the DRF places on the right side of the parabola and the promise of corticosteroids in patients requiring supplemental oxygen were described. Most importantly, placing COVID-19 in the context of the DRF can stimulate new thought concerning the pathogenesis of SARS-CoV-2 as a function of the host immune response. This provides a dynamic approach to research and therapy of this complex disease based on consideration of how to move patients’ immune responses from a state of disease to one of health.
